# The history of the CATH structural classification of protein domains

**DOI:** 10.1016/j.biochi.2015.08.004

**Published:** 2015-12

**Authors:** Ian Sillitoe, Natalie Dawson, Janet Thornton, Christine Orengo

**Affiliations:** University College London, Darwin Building, Gower Street, WC1E 6BT, UK

**Keywords:** Protein structure, Structure classification

## Abstract

This article presents a historical review of the protein structure classification database CATH. Together with the SCOP database, CATH remains comprehensive and reasonably up-to-date with the now more than 100,000 protein structures in the PDB. We review the expansion of the CATH and SCOP resources to capture predicted domain structures in the genome sequence data and to provide information on the likely functions of proteins mediated by their constituent domains. The establishment of comprehensive function annotation resources has also meant that domain families can be functionally annotated allowing insights into functional divergence and evolution within protein families.

## Highlights

• We present a historical review of the protein structure database CATH.
• We review the expansion of the CATH and SCOP resources with sequence data and functional annotations.
• How functional annotation resources allow insights into functional divergence and evolution within protein families.

## 1 Historical background

The major structural classifications, SCOP and CATH, were established in the mid-1990s. Several studies had shown the extent to which protein structures are conserved during evolution which suggested that 3D structure was a valuable fossil capturing the essential features of an evolutionary protein family and making it possible to identify even very remotely related proteins through similarities in their structures.

The first protein structure, myoglobin, was solved in 1958 and for the following three decades the number of structures solved and deposited in the Protein Databank [1] (PDB) only grew to be in the low thousands. At the time that the CATH and SCOP databases were established there were only ∼3000 protein structures in the PDB. Currently, in 2015, there are over 100,000.

**Fig. 1:**
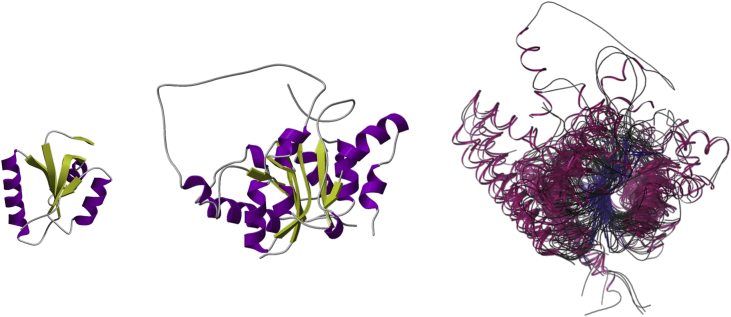
This figure shows the smallest (left figure) and largest (middle figure) domain structures from the “Nitrogenase molybdenum iron protein domain” CATH superfamily (ID: 3.40.50.1980) and a superposition of all non-redundant structural relatives from that superfamily (non-redundant at 35% sequence identity) (right figure). The superposition shows that structural ‘core’ of related protein structures can remain highly conserved even after the amino acid sequence has changed beyond recognition.

**Fig. 2:**
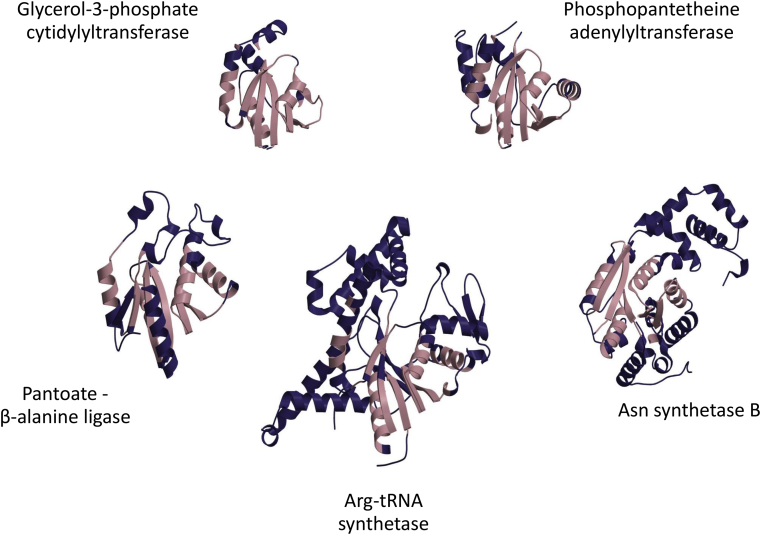
Selected relatives from the HUP superfamily (CATH ID: 3.40.50.620) illustrating the diverse structural embellishments (shown in blue) that have evolved and are embellishing the conserved structural core (shown in pink).

Although global structural characteristics (ie the folds of homologous proteins) are largely conserved during evolution [2], it is the buried secondary structures in the core of the protein domains that are most highly conserved (see Fig. 1) [3], [4]. Studies comparing protein domains showed that in more remote relatives, especially those from very distant species, there can be considerable insertions/deletions of amino acid residues [5]. These usually occur in the loops connecting the core secondary structures and can be very extensive, sometimes folding into additional secondary structures that decorate or embellish the structural core of the domain (see Fig. 2) [5].

The development of structure comparison algorithms by Rossmann and Argos [6] and Matthews and Remington [7] in the 1970s prompted several large scale analyses of protein structures and in 1976 Levitt and Chothia published a seminal paper which classified proteins according to their dominant secondary structure composition [8]. Four classes were recognized: mainly alpha-helical, mainly beta-strand, alternating alpha–beta and alpha plus beta structures. Other analyses by Thornton and Sternberg recognised common motifs recurring in particular classes. For example the right-handed beta–alpha–beta motifs recurrent in alpha–beta proteins [9] and different classes of beta-turns [10], [11].

Pioneering studies of groups of related proteins, by Chothia and Lesk [2], [12], [13], had also identified common structural features across homologous structures and described the variations (eg changes in loop lengths and secondary structure orientations) emerging with decreasing levels of sequence similarity. In the 1980s, their studies of the globins [2] and immunoglobulins [14] revealed highly conserved common cores detected in all relatives in these superfamilies despite almost undetectable levels of sequence similarity between the remote homologues ie in the midnight zone of <25% identity.

Lesk and Chothia also published a seminal study on the relationship between sequence identity and structural similarity [15] within homologous superfamilies which is still used to guide structure prediction and protein family assignment. This confirmed the incredible conservation of protein structure over massive evolutionary distances further supporting the notion that 3D structure could be exploited as an ancient fossil to detect family relationships.

These studies prompted the question of whether other general trends could be gleaned from reviews of protein families. Furthermore, the concurrent expansion of the PDB and development of more robust techniques for comparing structurally distant homologues led several groups to begin classifications of protein families based on structure. The domain was the focus of these studies as it was clear this represented the primary unit of evolution and proteins evolved through duplication, shuffling and fusion of domain sequences in genomes [16], [17].

Early structure comparison algorithms exploited rigid body algorithms to superimpose the structures based on the 3D coordinates and the methods struggled to achieve an optimal superimposition between distant homologues. Generally, they failed to converge on a solution where substantial insertions/deletions (indels) and changes in secondary structure orientations had occurred. Therefore in the late 1980s, more sophisticated approaches were explored (SSAP [18], COMPARER [19], DALI [20] discussed in more detail below) which used a variety of strategies for handling the shifts in secondary structure orientations and the extensive indels between distant homologues. These more robust approaches enabled large scale comparisons and classification of structural relatives.

The fortuitous development of the internet meant that classification data could be disseminated over the web. The SCOP [21] and CATH [22] classifications were the first to emerge in 1995, the former largely based on manual evaluation of structural similarities and the latter based on the SSAP algorithm, but these were followed by several other classifications using different automated structure comparison approaches for recognizing homologues. For example the COMPARER [19] method used by the Blundell group was applied to establish the HOMSTRAD resource [23] and the STAMP method used by the Barton group [24] to establish the 3DEE resource [25]. At the same time, other groups applied these approaches to recognizing structural neighbours. For example the DALI algorithm of Holmes and Sander was used to establish the DDD resource [26].

This chapter will focus on the evolution of the CATH domain structure classification which, together with the SCOP database, has endured and remains comprehensive and reasonably up-to-date with the now more than 100,000 protein structures in the PDB. We will review the expansion of the CATH resource to capture predicted domain structures in the genome sequence data and to provide information on the likely functions of proteins mediated by their constituent domains. The establishment of comprehensive functional resources, such as the Gene Ontology (GO [27]) has also meant that domain families in CATH can be functionally annotated allowing insights into functional divergence within protein families, which we will briefly discuss. Where appropriate, we will highlight similarities and differences in concepts used to establish and maintain the other widely used and comprehensive structural classification, SCOP.

## 2 Structural approaches used to recognize fold similarities and homologues

In the late 1980s the groups of Willie Taylor at NIMR and Tom Blundell at Birkbeck College London adapted the dynamic programming algorithms used to handle residue insertions and deletions in sequence alignment, to cope with the associated structural variations that these give rise to in 3D. Sali and Blundell extended this strategy to compare a range of features between proteins and used a Monte-Carlo optimization to obtain a structural superposition of relatives. This was encoded in the COMPARER algorithm [19]. Whilst Taylor and Orengo decided to employ a double dynamic strategy to go from 2D to 3D alignment and in 1989 developed the SSAP algorithm [18] which compared 3D views between residues in the proteins being compared and used a summary level to accumulate all the dynamic programming alignment ‘paths’ ie obtained by comparing ‘3D views’ from similar structural contexts. A final application of dynamic programming to this summary level determined the optimal alignment of the structures.

SSAP was demonstrated to be robust enough to cope with significant variations between homologues and revealed interesting ancestral relationships such as between the globins and plastocyanins that were undetectable using solely sequence data. Although the algorithm is relatively slow ie compared to DALI [20], COMPARER [19] and the more recent STRUCTAL [28], [29] and FATCAT [30] algorithms, this was not problematic in the mid 80s when there were fewer than 2000 protein structures in the PDB (a faster version of the method is now available: CATHEDRAL [31]).

**Fig. 3:**
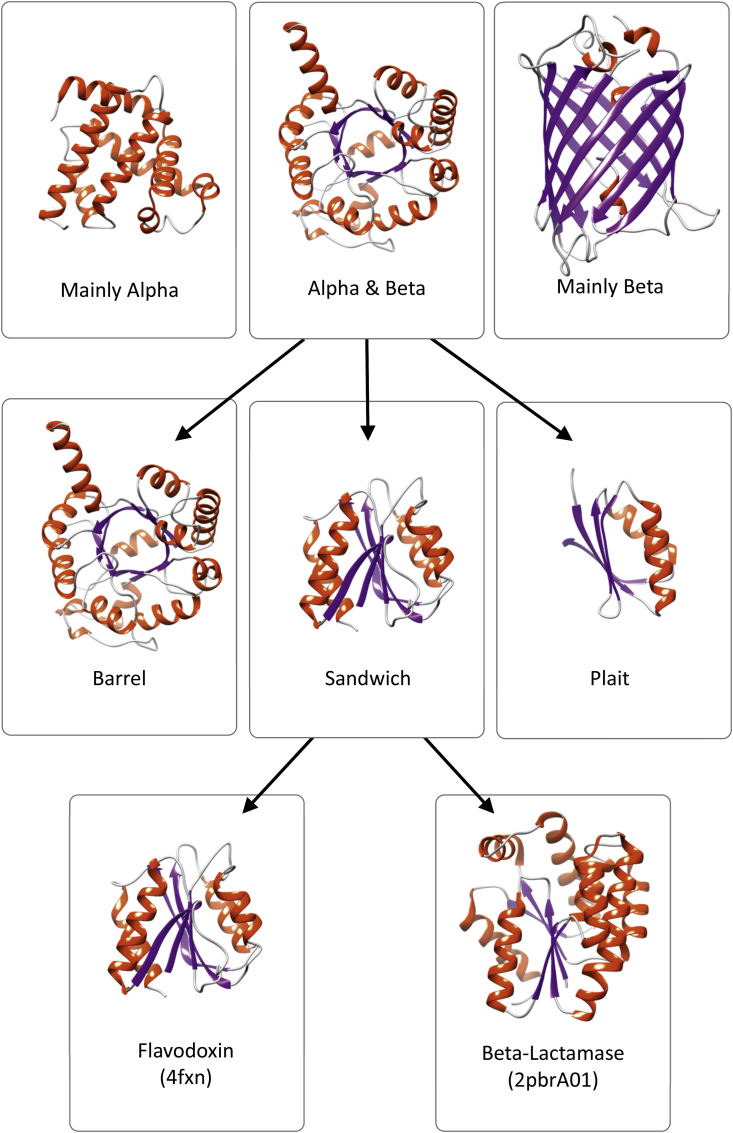
The first three levels of the CATH structure classification hierarchy: Class (based on secondary structure content), Architecture (based on gross spatial arrangement of secondary structures), Topology or Fold (similar folding arrangement of secondary structures).

Around this time, Janet Thornton, an expert in detailed studies of protein structure who had characterized various structural motifs like the alpha–beta-motifs [32], beta-turns [10], [11], recognised the potential of these more powerful comparison algorithms for classifying protein structures. She was keen to develop a classification system, similar to the Enzyme Commission approach used to describe enzymes, to capture the different types of folds and group together those that were similar. Orengo moved to the Thornton lab in the early 1990s and in 1993, Orengo and Thornton published a preliminary classification of around 1400 proteins structures based on the application of SSAP to detect homologues and proteins having similar folds [33]. The domain superfamilies identified in this way were further grouped into architectures where their secondary structures had similar orientations in 3D, and classes as defined by Chothia and Levitt ([8] and see above) (see Table 1 and Fig. 3).

As well as revealing global similarities, all against all SSAP comparison of structures in the PDB also revealed extensive local similarities [34] as many proteins, particularly in the mainly-beta and alpha–beta classes, comprise common recurrent secondary structure motifs resulting in extensive matches based on favoured arrangements of secondary structures in these classes.

Since a major aim was to report similarities reflecting evolutionary relationships or common folding constraints, the classification focused on global similarities where at least 60% of the larger protein could be well superimposed on equivalent residues in the smaller protein. Clustering of structures based on these criteria resulted in less than 1000 structural groups, described as ‘fold groups’ in which relatives were significantly similar in their structural cores [35].

It was clear from analyses of the data and relevant studies in the literature that some proteins adopting similar folds shared no other features indicative of an ancestral relationship ie no similarity in sequence motifs or functional properties and were therefore likely to be related through convergent rather than divergent evolution. In fact, given the physical constraints on packing alpha-helices and beta-sheets it is likely that there are a limited number of folding arrangements possible in nature. In order to identify homologous relationships, structurally similar domains were further analysed for sequence or functional similarity. Whilst close homologues (>=30% sequence identity) could easily be confirmed using pairwise algorithms like BLAST [36] or SSEARCH [37] for more distant homologues manual evaluation was required to examine functional similarities involving detailed visual inspection to detect shared and rare structural features and substantial reviewing of available literature.

This was a considerable task but arguably more reliable than relying entirely on completely automated approaches. Over the last decade or more, much more sophisticated sequence comparison techniques have been developed that can confirm homology even in the midnight zone of sequence similarity (<20% sequence identity). These are discussed in more detail below.

In 1997 the SSAP algorithm was modified to increase the speed for large scale comparisons within the PDB, by employing a filter that only allows comparison of proteins having sufficiently similar secondary structure arrangements and connectivity in their common structural core [31]. This new approach – CATHEDRAL – is nearly 1000 times faster than SSAP allowing CATH to remain up to date with the PDB.

The SCOP classification was largely constructed using manual evaluation of domain relationships although available algorithms such as BLAST [36] and DALI [20] were sometimes employed to guide this process. Despite the different approaches used between CATH and SCOP (ie largely manual for SCOP and semi-automatic using SSAP followed by manual curation for CATH) the two classifications identify similar numbers of fold groups and homologous superfamilies and comparisons between SCOP and CATH show a reasonable degree of equivalence between these structural groupings [38].

Both SCOP and CATH further classified the domain superfamilies and fold groups according to their architecture, where architecture describes the orientation of the secondary structure elements in 3D regardless of their connectivity. However, in CATH this was a formal level in the hierarchy whilst in SCOP architecture was simply an annotation. Finally domains were assigned to protein classes depending on the composition of secondary structure elements ie whether they were all-alpha, all-beta, or mixtures of alpha and beta (see Fig. 3) SCOP used more classes than CATH to capture these divisions but most domains fall into similar categories in the two classifications.

## 3 Domain recognition

Perhaps a major philosophical difference between the CATH and SCOP classifications is in the approaches used to identify domains within multi-domain protein structures. Domain recognition is problematic in that no formal quantitative definition of a domain exists. However, heuristic approaches search for compact, globular units with hydrophobic cores and more contacts between residues within the domain unit than between domain units. Furthermore secondary structures are unlikely to be shared between domains. These physical criteria have been encoded in a wide range of different algorithms since the 1990s. To identify domains in CATH, three independent such *ab-initio* methods (PUU [39], DETECTIVE [40], DOMAK [41]) based on these concepts are applied and the results compared to guide manual assignment of domain boundaries.

Accurately recognizing domain units using a purely algorithmic approach can be difficult, especially in large complex multi-domain proteins (ie comprising 4 or more domains). This is because the linking regions between domains can be quite small and the domain interfaces quite large and complex, especially if one of the domains is dis-contiguous. Therefore, it is difficult to optimize parameters in a way that doesn't lead to under-chopping or over-chopping of large complex multi-domain proteins. The difficulty in capturing the heuristic rules defining domains in an algorithm is illustrated by the fact that an analyses of the performance of these methods, judged using a manually validated benchmark set, showed that all three approaches only agreed about 10% of the time and that frequently there were considerable differences in the boundaries assigned [42]. For this reason expert curation is employed in ambiguous cases.

SCOP employs the same physical criteria in recognising domains by expert curation. Furthermore, a putative domain must be found to occur in at least two different independent contexts ie with different domain partners. This means that some protein regions deemed to be domains in CATH are not recognized as such by SCOP until additional data confirms the existence of this domain fused to different domain partners.

In 1995 both CATH and SCOP publicly launched their classifications via the web [35], [43]. Thus making it possible for biologists to browse through the data and view representative structures within each fold group and superfamily using the powerful new 3D visualization tool, Rasmol [44]. Both resources became widely adopted by both experimental and computational biologists with currently more than 10,000 unique visitors per month accessing the CATH and SCOP webpages.

## 4 Superfolds and the likely existence of limited folding arrangements in nature

**Fig. 4:**
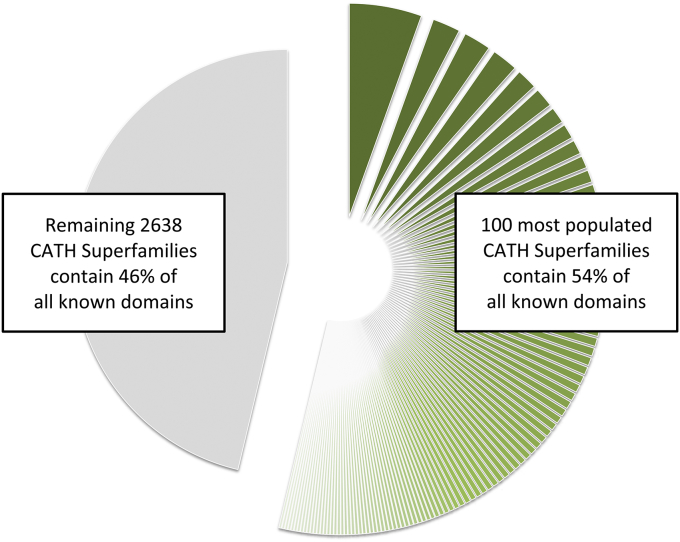
Plot showing the population of sequences in CATH domain superfamilies. More than half of all known protein domains in the genome sequences come from a small number (<5%) of highly populated superfamilies.

Perhaps the most interesting revelation to emerge from the structural classification data was the highly uneven distribution observed in the populations of the fold groups. In 1994 Orengo, Jones and Thornton reported the existence of ten ‘superfolds’ accounting for nearly 50% of all domain relatives in CATH [45]. Fig. 4 shows the percentage of non-redundant CATH domains currently assigned to the most highly populated superfamilies. Many of these adopt TIM barrel, Rossmann and other folds which possess very regular architectures ie layers of beta-sheets and/or alpha-helices. This regularity could be one factor explaining their frequent occurrence in nature. For example, these arrangements might be expected to accommodate mutations more easily because secondary structures would be more able to slide relative to each other, meaning that changes in residue size would be less likely to disrupt the core packing arrangements. Furthermore the large central super-secondary features eg beta-sheets or beta-barrels provide a stable core. Theoretical analyses have also suggested that these folding arrangements would be able to support large numbers of diverse sequences [46].

Based on the number of diverse sequence families found across the SCOP classification and the proportion of all known sequence data that this represented, Chothia postulated that there could be fewer than 1000 fold groups in nature [47], a relatively small number compared to the tens of thousands of known proteins at that time and therefore an exciting hypothesis which suggested that the use of structural classifications would make an understanding of protein evolution tractable.

In fact these predictions appear to have been largely borne out by the current data. Twenty years on, there are still only 1300 folds identified in CATH, and sensitive structure prediction tools suggest that CATH and SCOP capture nearly 80% of all protein domains in completed genomes with the remaining 20% being largely membrane associated domains which are likely to adopt relatively few diverse structural arrangements because of the physical constraints imposed by their location. The remaining sequences suggest highly disordered proteins that are not expected to adopt globular folds.

The structural classification data and large scale comparisons of domain structures also revealed novel folding motifs (split beta–alpha–beta motifs) common to a large proportion of alpha–beta domain superfamilies in which structures comprise a central antiparallel beta-sheet covered by a layer of alpha-helices (alpha–beta-plait folds [48]). Superfamilies adopting this fold contain one or two of these ‘split beta–alpha–beta-motifs’ [48]. They resemble the very common alpha–beta motifs earlier reported by Thornton and Sternberg [9], in which two parallel strands are connected by an alpha-helix, but in the ‘split beta–alpha–beta-motifs’ the beta-strands are effectively split by the third antiparallel beta-strand which hydrogen bonds to them both.

Development of faster structural comparison algorithms like GRATH [49] and CATHEDRAL [31] also allowed more extensive structure comparisons *between* protein superfamilies and suggested that relationships across CATH could be represented as a structural continuum due to similarities in the local folding motifs (eg alpha–beta; beta–beta and alpha–alpha motifs) a hypothesis that had been speculated as a ‘*russian doll effect*’ in the original CATH classification [35]. These analyses were confirmed by analyses from other groups [50], [51] and more recent analyses suggest that the extent to which a continuum exists depends on the region of structural architecture space you examine. In some regions, eg highly populated architectures in the alpha–beta class, fold space is very continuous, whilst in other regions more discrete fold islands exist [52]. Furthermore, some studies reveal that structural divergence within superfamilies can occasionally result in relatives possessing somewhat different folds [53]. CATH has dealt with this by providing information on structural relatives for each domain, whilst SCOP has recently launched a new resource SCOP2 that captures these interesting relationships in more detail [54].

## 5 Expansion of SCOP and CATH with predicted structures to further explore the evolution of domains

The international genome initiatives which started in the late 1990s and exploited rapid sequencing techniques, enabled the completion of the human genome by the millennium, and resulted in an explosion of sequence data by the turn of the century. This expansion of the sequence data combined with much more sensitive tools for recognizing sequence similarities prompted the recruitment of sequence relatives into the domain superfamilies of SCOP and CATH. Both resources exploited powerful sequence profiles – known as Hidden Markov Models (HMMs) – which were built from multiple alignments of sequence clusters in domain superfamilies and used to recognize domain relatives in the genome sequences. Two sister resources were established – Gene3D [55] associated with CATH and SUPERFAMILY [56] associated with SCOP. In parallel, purely sequence based protein domain classifications were established (eg Pfam [57]) and these are currently integrated with Gene3D [55], SUPERFAMILY [56] and other resources (PRINTS [58], PANTHER [59], HAMAP [60], SMART [61]) in the widely used InterPro resource [62] at the EBI.

These strategies brought about an ∼100-fold increase in the number of domains assigned to CATH and SCOP allowing more detailed analyses of evolutionary relationships and in particular an understanding of the divergence of sequences and functions within particular superfamilies (discussed more below). Currently more than 50 million sequences are assigned to CATH superfamilies in Gene3D. SUPERFAMILY which has explicitly incorporated completed genome data not yet deposited in public repositories like UniProt and ENSEMBL, has more than 40 million sequences from 2500 completed genomes. This data is likely to expand further in the near future as the metagenome projects bring in millions more relatives from species in diverse environments from around the globe.

By recognizing CATH or SCOP domains within protein sequences it was possible to trace the emergence of novel proteins resulting from different domain fusions or fissions. For example, Vogel and Chothia examined changes in domain partnerships in proteins linked to the immunoglobulin superfamily. In worm, expansions in multi-domain architectures were linked to the expansion of structural proteins whilst in fly, expansions involving related domains enhanced the immune system repertoire [63]. Comprehensive studies of Gene3D showed that whilst nearly 70% of domain superfamilies were found in all kingdoms of life these were usually combined in different ways in the different kingdoms and species so that less than 10% of multi-domain proteins were common to all kingdoms of life [64]. Studies by Teichmann, Gerstein and others exploiting similar data from SCOP cite these phenomena as supporting a ‘mosaic theory of life’ [65]. However, the fact that some domain superfamilies recur much more frequently than others – the top 100 domain superfamilies (ie < 5% of all superfamilies) account for over 50% of all domains in CATH (see Fig. 4) – suggest that an alternative description might be of a ‘Lego theory of life’ where some common domains recur extensively in different contexts. Later analyses revealed a core set of about 200 highly populated domain superfamilies which could be traced back to the last common ancestor – LUCA [66].

## 6 Exploiting domain structure superfamilies in CATH to examine the evolution of protein functions

The expansion of CATH superfamilies with sequence data considerably increased the amount of functional data too, allowing large scale studies of the divergence of function within superfamilies during evolution. These studies showed that whilst relatives in most superfamilies shared a common function, in the most highly populated superfamilies considerable divergence of sequence and function had occurred. A detailed study of 31 such diverse superfamilies revealed the molecular mechanisms by which functions had changed [67]. These phenomena ranged from small local changes eg mutations of residues in the active site (which modified chemistry or substrate specificity) or insertions of residues around the active site (which largely affected binding of substrates); through to fusions of domains with different partners (which could in turn modify active site geometries). Mutations and residue insertions in other sites on the protein surface could bring about changes in protein interactions or changes in oligomerization state, again altering active site geometries.

Studies of enzyme superfamilies revealed that changes in function were usually associated with variations in the substrate specificity. Changes in chemistry were much less frequent presumably because it is harder to engineer a novel chemistry than to alter the binding site and change the compound on which that chemistry is performed [67]. By exploiting the sequence data and considering the distribution of superfamily relatives on metabolic paths Thornton and co-workers showed [68] that the data supported a ‘pathwork’ theory of evolution, originally proposed by Horwitz et al. [69] whereby relatives are generally recruited to metabolic paths to perform a particular chemistry. This was in contrast to a hypothesis suggested by Jensen [70] whereby relatives within a family were thought to be more likely to be found in the same pathway and were proposed to have diverged to make different steps along the reaction pathway more efficient.

More recent collaborations between the Thornton and Orengo groups have led to the establishment of a new resource, FunTree [71] in the Thornton group. This combines evolutionary data from CATH, presented as phylogenetic trees, with information on catalytic residues, substrates and chemical mechanisms for all enzyme superfamilies. Catalytic residue data and chemical mechanisms are integrated from the CSA [72] and MACIE [73] resources, respectively, both developed in the Thornton group.

FunTree enabled much more comprehensive studies of functional divergence in domain superfamilies and confirmed the previously observed trends of conservation of chemistry between parent and child nodes in the phylogenetic tree but also highlighted frequent divergence in substrate specificity within some promiscuous enzyme superfamiles. However, significant changes in chemistry can still occur [74].

As regards the structural mechanisms mediating functional change, analyses of some structurally and functionally divergent CATH superfamilies revealed substantial insertion of residues often resulting in additional secondary structural features packed against the common structural core of the domain and appearing as embellishments to that highly conserved core. In some large CATH superfamilies, relatives differ in size by three-fold in the number of residues or more [75]. Detailed studies of the large and highly promiscuous HUP domain superfamily in CATH showed that indels and the resulting secondary structure embellishments were distributed along the entire length of the polypeptide chain. However, since these indels were constrained to loops between core secondary structures and because of the general architectural features of the domain (ie with a central beta-sheet), these tended to accumulate in relatively few positions on the protein surface. In particular, they aggregated around the active site pocket situated at the top of the beta-sheet (where they modify substrate specificity) and in other surface sites where they alter interactions with domain and protein partners [76].

## 7 Functional sub-classification in CATH-Gene3D and what this reveals about the evolution of enzyme active sites

**Fig. 5:**
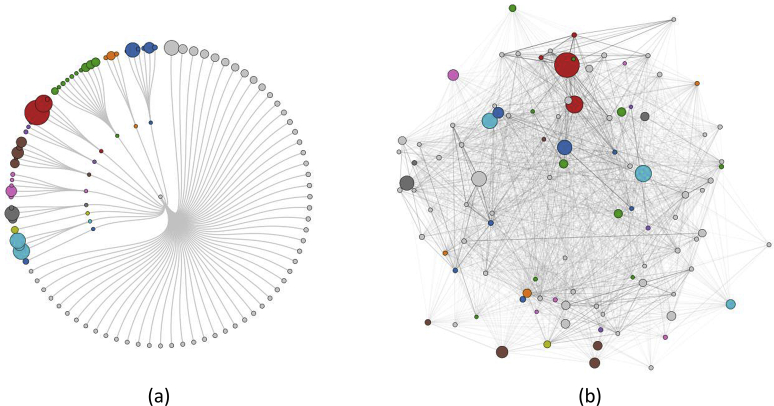
Functional Families (FunFams) in CATH aim to cluster protein domains that all share a specific function. Relationships between FunFams within a superfamily can be visualised in a number of ways including a) clustering according to structural similarity and b) networks according to global sequence similarity.

More recently, the extreme divergence of functional properties of relatives in some highly populated CATH superfamilies prompted the development of protocols to sub-classify superfamilies into functional families (termed FunFams, see Fig. 5). This was achieved using a profile based protocol that recognized differences in specificity determining residues between putative families [77]. This is a challenging task as it requires sufficient sequence diversity across a FunFam to enable detection of conserved residues. As a result, it is harder to distinguish functional groups having narrow species distribution and these groups will tend to merge with functionally close families. Nevertheless independent validation by an international assessment (CAFA [78]) showed the FunFams to be highly competitive in providing functional annotations.

CATH-Gene3D currently identifies 110,000 functional families within 2700 superfamilies. 360 of these superfamilies comprise a single FunFam. In contrast, 350 of the largest superfamilies account for 75% of the FunFams. These are large, universal superfamilies found in all kingdoms of life and accounting for more than 60% of all predicted domain sequences in CATH-Gene3D.

Sub-classification into functional families allows comparison of functional sites between relatives across a superfamily and gives insights into evolutionary mechanisms underlying shifts in function. Information on functional sites is largely restricted to relatives of known 3D structure which on average comprise less than 10% of sequences within the superfamily (or less in some of the more ubiquitous and diverse superfamilies). Comparisons of interfaces across superfamilies showed that functionally diverse relatives were exploiting different surface patches on the structure ie distinct sites, depending on their interaction partner, and that paralogues shared few common interactors. However, quite frequently there was one location that was more frequently exploited by diverse paralogues [5].

Recent analyses of changes in catalytic residues in different functional families across 101 well annotated CATH enzyme superfamilies showed that in the majority of superfamilies (∼70%) at least two FunFams had significant differences in their catalytic machineries (ie the nature and location of catalytic residues in the active site showed less than 50% similarity between the relatives). Unsurprisingly, most of the time these led to changes in the chemistries of the relatives or the substrates being acted upon. However, in 25% of the cases examined the same chemistry was associated with completely different catalytic machineries. This may be a consequence of evolutionary drift from a common ancestor with a particular function, resulting in modified efficiency in one relative which is then compensated by further mutations to optimize the physico-chemical features of the active site [79]. Alternatively, it may imply convergent evolution within the superfamily as in the Rubisco superfamily where a more efficient form of this important protein responsible for carbon fixation, has emerged more than 60 times during evolution [80].

## 8 Conclusions

The SCOP and CATH classifications organize the 3D structure of proteins into evolutionary classifications that have enabled detailed studies of the molecular mechanisms by which new protein structures and functions evolve. The sequence patterns and fold libraries that they provide have enabled prediction of structural relatives thereby providing structural annotations for more than 50 million domain sequences, available on their sister sites (Gene3D, Superfamily respectively) and in InterPro [62]. The predicted data revealed the power law bias in superfamily populations whereby most superfamilies are small but a few hundred are universal and very highly populated [81], [82]. Combination of the sequence and structure data have supported large scale comparative genome studies which revealed changes in domain architecture across different species modifying the functional repertoires of those species [2], [14]. They have also enabled phylogenetic studies that traced the evolution of different chemistries within enzyme superfamilies [74]; and structural studies that revealed the changes in the catalytic machineries that bring about these functional shifts [79]. CATH superfamilies have also been used to detect patterns of domain presence and absence in genomes that allow predictions of protein interactions [83].

Although ∼20–25% of domain sequences in the genomes do not currently map to any structural superfamilies in CATH or SCOP, the analyses of structures solved by the structural genomics initiatives in the States – which targeted structurally uncharacterized domain families in Pfam for structural determination – showed that once the structures of these superfamilies had been solved they revealed a structural or evolutionary relationship with an existing fold group or superfamily in SCOP or CATH. In fact, nearly 98% of all new structures deposited in the PDB can be classified in an existing CATH superfamily [25], suggesting that these classifications now account for the majority of superfamilies in nature.

Over the last few years collaborations between SCOP and CATH have led to mappings between these resources that help to confirm detection of very remote homologues [38]. Future collaborations are likely to enhance the quality of the data in both resources by removing errors and sharing curation tasks to enable these resources to keep pace with the still exponential increases in the structure and sequence data.

## Figures and Tables

**Table 1:** A summary of the terms used in common between the CATH and SCOP structure classification databases.

CATH	SCOP	Description
Class	Class	Hierarchy separated by gross structural differences (e.g. secondary structure content)
Architecture	–	Similar general organization of secondary structures within 3D space
Topology (fold)	Fold	Structural similarity without clear evidence of evolutionary similarity
Homologous superfamily	Superfamily	Structural and functional features suggest a common evolutionary origin (often despite low sequence similarity)
–	Family	Clusters domains with clear evolutionary relationship (usually including significant sequence similarity)
FunFam	–	Clusters domains with functional similarity
